# Author Correction: Single cell transcriptomic profiling of a neuron-astrocyte assembloid tauopathy model

**DOI:** 10.1038/s41467-026-77856-8

**Published:** 2026-09-21

**Authors:** Hannah Drew Rickner, Lulu Jiang, Rui Hong, Nicholas K. O’Neill, Chromewell A. Mojica, Benjamin J. Snyder, Lushuang Zhang, Dipan Shaw, Maria Medalla, Benjamin Wolozin, Christine S. Cheng

**Affiliations:** 1 Department of Biology, Boston University, Boston, MA 02215 USA; 2 Department of Pharmacology and Experimental Therapeutics, Boston University School of Medicine, Boston, MA 02118 USA; 3 Program in Bioinformatics, Boston University, Boston, MA 02215 USA; 4 Department of Anatomy & Neurobiology, Boston University School of Medicine, Boston, MA 02118 USA; 5 Informatics Group, J. Craig Venter Institute, La Jolla, CA 92037 USA; 6 Department of Neurology, Boston University School of Medicine, Boston, MA 02118 USA; 7 Center for Systems Neuroscience, Boston University, Boston, MA 02118 USA; 8 Department of Psychiatry, University of California San Diego, La Jolla, CA 92093 USA

**Keywords:** Alzheimer's disease, Astrocyte, Neural stem cells

Correction to: *Nature Communications* https://doi.org/10.1038/s41467-022-34005-1, published online 21 October 2022

The original version of this article, contained an error in Fig 6, in which the representative images in Fig 6b, row ‘oTau + Vehicle’, were inadvertently copied from Fig 2d showing representative images of the oTau in row ‘63x’.

The correct version of Fig 6 is:

**Figure:**
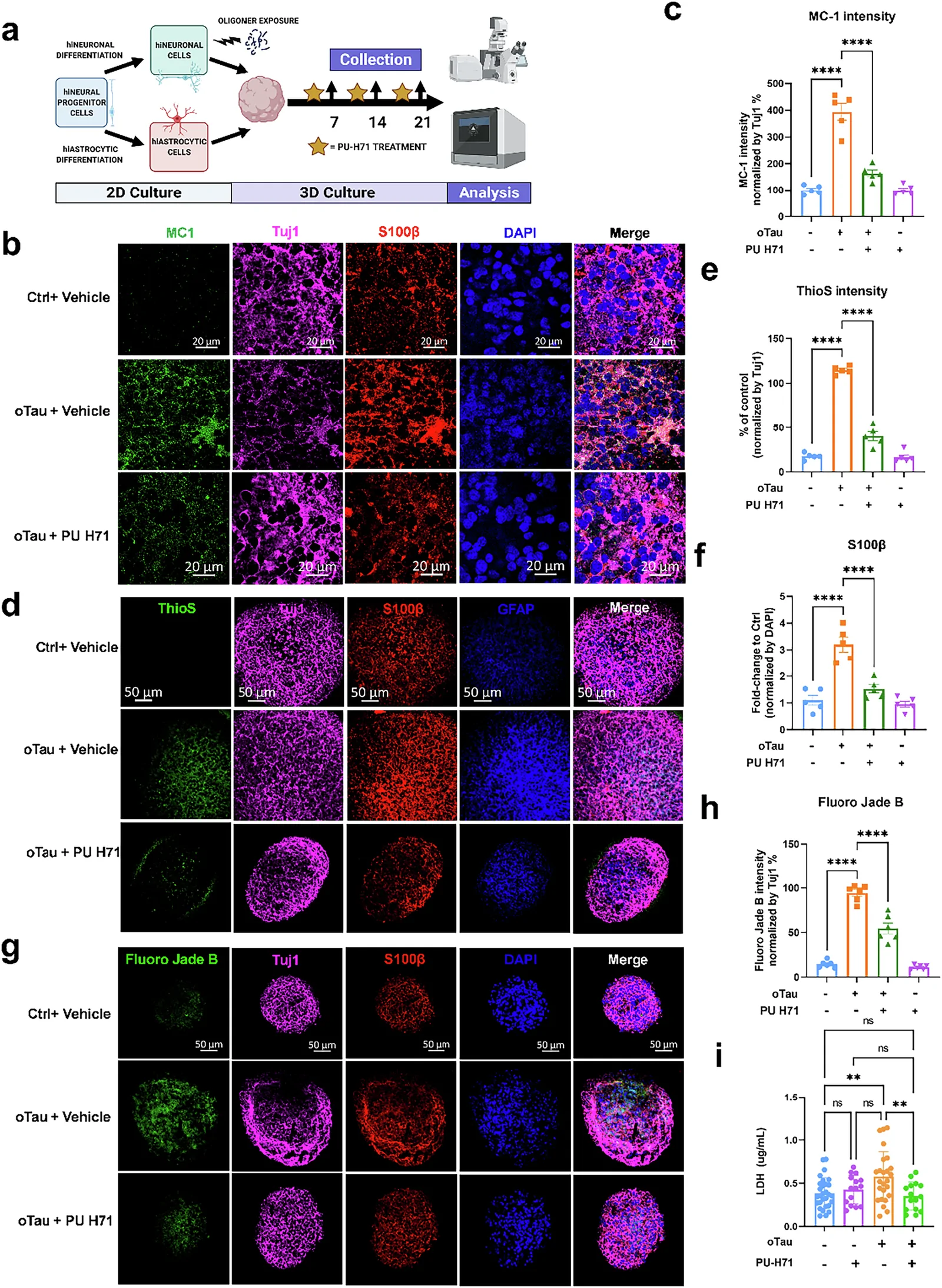

which replaces the previous incorrect version:

**Figure:**
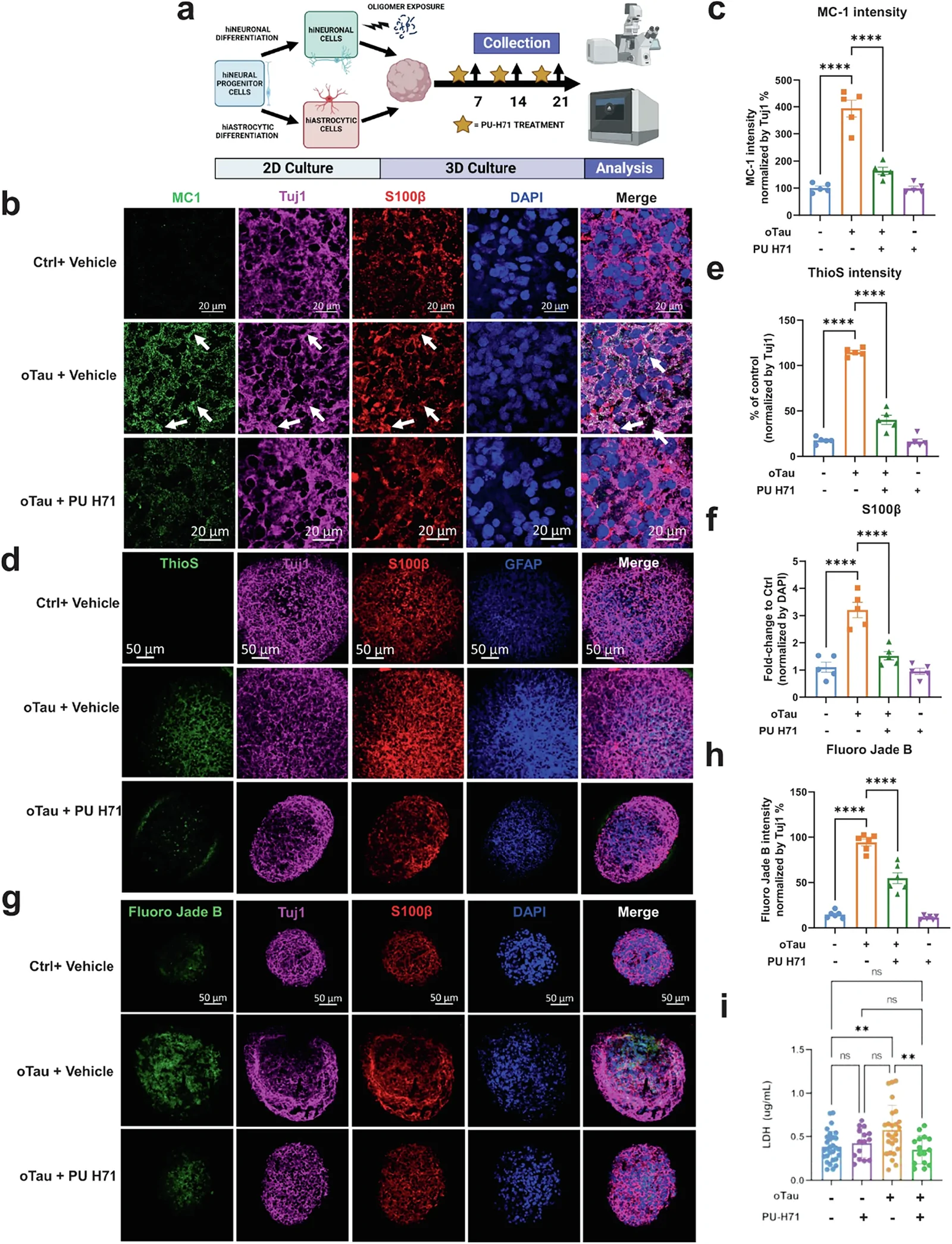

This has been corrected in both the PDF and HTML versions of the article.

