# Supplementary material for: Single cell transcriptomic profiling of a neuron-astrocyte assembloid tauopathy model
Source: Nat Commun. 2022 Oct 21;13:6275. doi: 10.1038/s41467-022-34005-1 (PMC9587045; doi:10.1038/s41467-022-34005-1)
Supplement: Supplementary file 3 — Reporting Summary [file 41467_2022_34005_MOESM3_ESM.pdf]

nature research

Corresponding author(s): Christine Cheng

Last updated by author(s): Oct 7, 2022

Reporting Summary

Nature Research wishes to improve the reproducibility of the work that we publish. This form provides structure for consistency and transparency in reporting. For further information on Nature Research policies, see Authors & Referees and the Editorial Policy Checklist .

Statistics

For all statistical analyses, confirm that the following items are present in the figure legend, table legend, main text, or Methods section.

n/a	Confirmed	
☐	☒	The exact sample size (*n*) for each experimental group/condition, given as a discrete number and unit of measurement
☐	☒	A statement on whether measurements were taken from distinct samples or whether the same sample was measured repeatedly
☐	☒	The statistical test(s) used AND whether they are one- or two-sided<br>*Only common tests should be described solely by name; describe more complex techniques in the Methods section.*
☐	☒	A description of all covariates tested
☐	☒	A description of any assumptions or corrections, such as tests of normality and adjustment for multiple comparisons
☐	☒	A full description of the statistical parameters including central tendency (e.g. means) or other basic estimates (e.g. regression coefficient) AND variation (e.g. standard deviation) or associated estimates of uncertainty (e.g. confidence intervals)
☐	☒	For null hypothesis testing, the test statistic (e.g. *F*, *t*, *r*) with confidence intervals, effect sizes, degrees of freedom and *P* value noted<br>*Give P values as exact values whenever suitable.*
☒	☐	For Bayesian analysis, information on the choice of priors and Markov chain Monte Carlo settings
☒	☐	For hierarchical and complex designs, identification of the appropriate level for tests and full reporting of outcomes
☐	☒	Estimates of effect sizes (e.g. Cohen's *d*, Pearson's *r*), indicating how they were calculated

Our web collection on statistics for biologists contains articles on many of the points above.

Software and code

Policy information about availability of computer code

Data collection	10X Genomics Chromium 5.00<br>SoftMax Pro 7.1 Software<br>Agilent 2100 BioAnalyzer Expert Software<br>Illumina NextSeq 500 Software<br>Illumina NOVA-seq Software<br>Carl Zeiss Confocal LSM700 Software<br>Zeiss microscope Zen black LSM700 and LSM880<br>ProteinSimple SimpleWestern Jess Software<br>uMs-Nikon FN1 infrared-differential interference contrast (IR-DIC) microscope software
Data analysis	ImageJ/FIJI 1.53c<br>GraphPad Prism version 9.00 for Windows<br>10X Genomics CellRanger v3.1.0<br>STAR Aligner v2.7<br>Cytoscape 3.8.2<br>EnrichmentMap v3.3.4<br>R v4.0.3<br>Seurat R package v4.1.1<br>SingleR R package v1.2.4<br>ComplexHeatmap R package v2.4.3<br>GProfiler2 R package v0.2.0<br>ggplot2 R package v3.3.2<br>Circlize R package v0.4.15

Liger R package v1.0.0
Monocle3 R package v1.0.0
Enrichplot R package v1.10.2
DOSE R package v3.16.0
All custom code to reproduce the analyses and figures reported in this paper can be found at DOI: 10.5281/zenodo.7102480

For manuscripts utilizing custom algorithms or software that are central to the research but not yet described in published literature, software must be made available to editors/reviewers. We strongly encourage code deposition in a community repository (e.g. GitHub). See the Nature Research guidelines for submitting code & software for further information.

Data

Policy information about availability of data

All manuscripts must include a data availability statement. This statement should provide the following information, where applicable:

- Accession codes, unique identifiers, or web links for publicly available datasets
- A list of figures that have associated raw data
- A description of any restrictions on data availability

The authors declare that all data supporting the findings of this study and associated accession codes for publicly available datasets are available within the paper [and its supplementary information files].
Raw and processed scRNA-seq data generated in this study has been deposited in the NCBI Gene Expression Omnibus database (GEO) under accession code GSE186356 [https://www.ncbi.nlm.nih.gov/geo/query/acc.cgi?acc=GSE186356]. Processed scRNA-seq datasets generated in this study have been deposited on Single Cell Portal, including the cell barcodes, UMAP coordinates, and other available characteristics [https://singlecell.broadinstitute.org/single_cell/study/SCP1621/asteroid1-2021].
Publicly available dataset used in this study are available at The Molecular Signatures Database [https://www.gsea-msigdb.org/gsea/msigdb] and The Human Primary Cell Atlas [http://biogps.org/dataset/2429/primary-cell-atlas/].
Source data are provided with this paper. Source data are provided in the Source Data files as follows: Source data of differential gene expression results for Fig. 4c and Fig 7b, cell type markers for Suppl. Fig. 5c, and uncropped immunoblots for Fig. 3c,h, GProfiler2 FGSA results and gene set names for Fig. 5a, Fig. 7e, Fig. 8g, Suppl. Fig. 14h, Suppl. Fig. 15, and Suppl. Fig. 16, GProfiler2 FGSEA results in Cytoscape formatting for Fig. 5b and Suppl. Fig. 17a,b, gene set names and GProfiler2 FGSEA results for Fig. 5c, and module scores for Fig. 8e and Suppl. Fig. 14e are available in supplemental file SourceFile. Detailed explanations of gene sets used Fig. 4d,e and Fig. 7 c,d are also available in supplemental file SourceFile.
The original R scripts for Seurat processing are available on GitHub [https://github.com/satijalab/Seurat]. All custom code to reproduce the analyses and figures reported in this paper is available on GitHub [DOI: 10.5281/zenodo.7102480]124.

Field-specific reporting

Please select the one below that is the best fit for your research. If you are not sure, read the appropriate sections before making your selection.

[x] Life sciences [ ] Behavioural & social sciences [ ] Ecological, evolutionary & environmental sciences

For a reference copy of the document with all sections, see nature.com/documents/nr-reporting-summary-flat.pdf

Life sciences study design

All studies must disclose on these points even when the disclosure is negative.

Sample size	No statistical method was used to predetermine sample size. Sample size determination for immunolabeling quantification was based on prior studies with 3D assembloid modeling28,32,37. High-throughput scRNA-seq was selected to profile <1000 cells per replicate experimental condition providing a sufficiently large sample size for analysis.
Data exclusions	No data were excluded from the immunolabeling analyses. Standard quality control filtration was performed in the analysis of the scRNA-seq data excluding cells with less than 200 and greater than 3000 detected genes or greater than 12% mitochondrial counts to remove low quality and multiplet cell reads.
Replication	All immunolabeling experiments were successfully repeated in at least 3 independent batches of asteroid cultures with at least 5 individual asteroids per quantification. scRNA-seq was successfully repeated in 4 independent batches of asteroid cultures.
Randomization	Cultured 3D asteroids within a batch were blindly and randomly selected at timepoint collection for immunolabeling or scRNA-seq. Additionally, well plates were randomized for treatment to avoid marginal effects of cell growth on the plate. Further covariate control is not relevant to this study due to the highly controlled nature of the culture system.
Blinding	Quantification of transcriptomics, granular intensity, MAP2 dendritic length, and immunoblot band intensity were blindly repeated by co-authors.

Reporting for specific materials, systems and methods

We require information from authors about some types of materials, experimental systems and methods used in many studies. Here, indicate whether each material, system or method listed is relevant to your study. If you are not sure if a list item applies to your research, read the appropriate section before selecting a response.

Materials & experimental systems

n/a	Involved in the study	
☐	☒	Antibodies
☐	☒	Eukaryotic cell lines
☒	☐	Palaeontology
☐	☒	Animals and other organisms
☒	☐	Human research participants
☒	☐	Clinical data

Methods

n/a	Involved in the study	
☒	☐	ChIP-seq
☒	☐	Flow cytometry
☒	☐	MRI-based neuroimaging

Antibodies

Antibodies used

For protein biochemistry: Tau13 (1:10,000, Davies Lab, Northwell), Tau5 (1:1000, mouse, Kanaan lab, MSU). Mouse Monoclonal phospho-Tau (Ser202, Thr205) antibody (AT8) purchased from Invitrogen catalog # MN1020 (RRID: AB_223647).

The primary antibodies used in this study for immuno-fluroscence are as follows: Chicken polyclonal anti-MAP2, 1:250 (Aves Labs, Cat#MAP, RRID: AB_2313549); Rabbit monoclonal anti-Cleaved Caspase 3 (Asp175) (5A1E), 1:400 (Cell Signaling Technology, Cat# 9664, RRID:AB_2070042); Mouse monoclonal phospho-Tau (Thr181) antibody AT270, 1:400 (Thermo Fisher Scientific, Cat# MN1050, RRID:AB_223651); Mouse monoclonal phospho-Tau (S262) antibody 12E8, 1:400 (provided by Philip Dolan, Prothena); Rabbit Polyclonal anti-hnRNP A2B1, 1:300 (Thermo Fisher Scientific, Cat# PA534939, RRID:AB_2552288); Mouse monoclonal anti-TOMA2, 1:300(provided by Rakez Kayed lab, University of Texas Medical Branch); VGLUT1 (Synaptic Systems, Cat#135-203, RRID:AB_887886), GAD67 (Thermo Fisher Scientific, Cat# PA5-19065, 1:100, RRID: AB_10987356). All the secondary antibodies were purchased from Jackson ImmunoResearch.

Validation

MAP-2, Tau-13, TOMA2, Cleaved Caspase 3, 12E8 antibodies were validated in our previous publications: PMID: 30465259, PMID: 30068389, PMID: 29273772. T181 pTau, hnRNPA2B1, VGLUT1 and GAD67 antibodies were validated by the manufacture as shown on their website.

Eukaryotic cell lines

Policy information about cell lines

Cell line source(s)

Human iPSC (XCL-1) derived neural progenitor cells (NPCs), Stem Cell Technologies cat#70901

Authentication

Cell lines were authenticated by Stem Cell Technologies.

Mycoplasma contamination

Mycoplasma contamination was tested showing negative result.

Commonly misidentified lines
(See ICLAC register)

Name any commonly misidentified cell lines used in the study and provide a rationale for their use.

Animals and other organisms

Policy information about studies involving animals; ARRIVE guidelines recommended for reporting animal research

Laboratory animals

PS19 mice overexpressing human P301S Tau (B6;C3-Tg(Prnp-MAPT*P301S)PS19Vle/J, stock #008169) were purchased from Jackson Laboratories. Male and female PS19 P301S tau+/- mice were used as breeding pairs and the F1 generation of P301S tau +/-(PS19) and P301S tau-/-(wild type) were used for the experiment. 12 mice (6male and 6 female) were used in this study. Mice were sacrificed for experiment at the age 9 months old for preparing Tau oligomers.

Wild animals

NA

Field-collected samples

NA

Ethics oversight

Use of animals was approved by the Boston University Institutional and Animal Care and Use Committee (AN15301, PROTO201800234). All animals were housed in IACUC-approved vivariums at Boston University School of Medicine.

Note that full information on the approval of the study protocol must also be provided in the manuscript.
